# Genetic diversity and population structure of *Haloxylon salicornicum* moq. in Kuwait by ISSR markers

**DOI:** 10.1371/journal.pone.0207369

**Published:** 2018-11-21

**Authors:** Fadila Al Salameen, Nazima Habibi, Vinod Kumar, Sami Al Amad, Jamal Dashti, Lina Talebi, Bashayer Al Doaij

**Affiliations:** 1 Biotechnology Program, Environment and Life Sciences Research Centre, Kuwait Institute for Scientific Research, Shuwaikh, Kuwait; 2 Desert, Agriculture & Ecosystems Program, Environment and Life Sciences Research Centre, Kuwait Institute for Scientific Research, Shuwaikh, Kuwait; 3 Environment Pollution and Climate Change Program, Environment and Life Sciences Research Centre, Kuwait Institute for Scientific Research, Shuwaikh, Kuwait; University of Helsinki, FINLAND

## Abstract

*Haloxylon salicornicum* moq. Bunge ex Boiss (Rimth) is one of the native plants of Kuwait, extensively depleting through the anthropogenic activities. It is important to conserve *Haloxylon* community in Kuwait as it can tolerate extreme adverse conditions of drought and salinity to be potentially used in the desert and urban revegetation and greenery national programs. Therefore, a set of 16 inter simple sequence repeat (ISSR) markers were used to assess genetic diversity and population structure of 108 genotypes from six locations in Kuwait. The ISSR primers produced 195 unambiguous and reproducible bands out of which 167 bands were polymorphic (86.5%) with a mean PIC value of 0.31. The overall average values of Nei’s gene diversity (*h'*) and Shannon’s diversity index (*I*) were 0.254 and 0.375, respectively. Results of AMOVA revealed high genetic variations within populations (77.8%) and low among populations (22%). The values of Fixation index (F_ST_ = 0.22; P = 0.0), Genetic differentiation (G_ST =_ 0.262; G’_ST_ = 0.327; D = 0.335 and Gene flow (N_M_ = 0.880) were indicative of heterozygous populations. The results of STRUCTURE and split decomposition analysis suggested that the Rimth accessions of Kuwait can be grouped into five and six subpopulations, respectively. Principal coordinate analysis (PCoA) grouped them into three clusters. The pairwise Nei’s genetic distances (D_S_) among populations demonstrated a narrow range from 0.047 to 0.187 (Scale-0.0 to 1.0). The Mantel’s test revealed a weak correlation (*r*^*2*^- 0.188; P-0.013) between the genetic distance and geographic distances. Our results suggest that the narrowly distributed *Haloxylon* community in Kuwait demonstrated a high genetic diversity within the populations however the overall population structure was weak.

## Introduction

Kuwait is an arid country with smallest land area. The native plants have a narrow distribution. The species that exist in the ecosystem are unique as they offer a valuable genetic pool for drought, heat and salt-tolerance [1]. In addition they have a potential for phyto-remediation, ornamented landscaping and erosion prevention. However, the size of the area containing plants is decreasing on account of environment degradation and climate change. Desertification is a world-wide problem, but intensified in arid zone as in case of Kuwait. Over 90% of the total land area suffers from desertification and 44% is very severely degraded. [2]. Human interventions such as off road driving, camping and impact of the Gulf war further pose a risk to the vegetative communities [3]. In order to conserve its native plant community, Kuwait became a signatory to the Convention on biological Diversity (CBD) [4] and has embarked on a major plan for restoration of several species.

*Haloxylon salicornicum* Moq. Bunge ex Boiss (Rimth) is one of the main structural elements in Eastern Arabian vegetation associations [1,5] belonging to the family Amaranthaceae. It is a perennial herb that is widely available in Egypt, Palestine, Jordan, Iraq, Iran, Pakistan and the northeastern part of Kuwait Bay and southern coastal areas [6,7]. The plant is utilized as food source for domestic stock and wildlife, stabilizes the soil surface besides providing suitable micro climate, camouflage and harbors various animals [1]. It is considered as one of the most promising species for revegetation and sand dune fixation [7]. *H*. *salicornicum* community is under threat from overgrazing leading to reduction in percentage of distribution from 22.7% to 2.2% in Kuwait [8,9]. The populations from Southern borders have retreated to a considerable extent and the existing populations can only be found along the North East borders. Studies on morphological diversity of *Haloxylon* communities demonstrated considerable variations due to wind erosion and land degradation [6]. Information on genetic diversity and population structure is therefore, desired for this community to aid the conservation and restoration programs being undertaken [10].

To formulate effective conservation and restoration strategies, the understanding of a population’s genetic diversity is imperative. A wide range of molecular methods are now being applied to measure the genetic diversity of a population. The long-term survival of a species is challenging particularly if it has reduced variability. These species suffer from homozygosity and inbreeding depression causing reduction in reproduction rates indirectly leading towards extinction [11]. A narrow population faces the risks of genetic drifts, complete gene migration resulting in fatal consequences. Assessment and preservation of biodiversity of small populations is therefore, crucially important to minimize the loss of initial genetic variation as a consequence of, inbreeding or genetic drifts [12]. In addition to this the information on genetic structure, genetic diversity and genetic differentiation provides information on selection of propagules for establishing new populations.

In recent years, PCR based molecular markers has allowed the use of DNA sequences in genetic analyses and has provided, a better understanding of the genetic diversity and differentiation of natural populations [13]. Molecular markers have been widely used as variability indicators by comparing the individual species at several polymorphic loci. The first use of ISSR primers began in the early 90s [14–16]. Microsatellites are distributed throughout the Eukaryotic genome as short repeats of 2 to 5 bp length. ISSRs are present between the repetitive microsatellites. Primers complementary to microsatellite regions randomly amplify the genomic DNA that can be used to evaluate genetic variation. ISSR is an extensively employed technique in the detection of genetic variations due to its many advantages such as quick performance, cost effectiveness, low quantity of template and no prior knowledge about the target sequences [17]. A good genetic marker is defined by its high rate of polymorphism and the ability to generate multi-locus data from the genome under study. The ISSR markers make use of microsatellite sequences that are inherently highly variable and ubiquitously distributed across the genome, at the same time achieving higher reproducibility as compared to other genetic markers [18,19]. ISSR DNA markers have been widely applied in genetic characterization of desert plant species [20–23]. ISSRs have also been used as a technique to establish conservation strategies for endangered plants such as *Agave victoriae* [24], *Acacia raddiana* [25] and *Breonadia salicina* [26].

In view of the aforementioned the aim of this study was to assess the genetic diversity and population structure of *H*. *salicornicum*. This would aid in defining conservation studies for the declining *Haloxylon* in Kuwait.

## Materials and methods

### Collection site and sample collection

**Fig 1:**
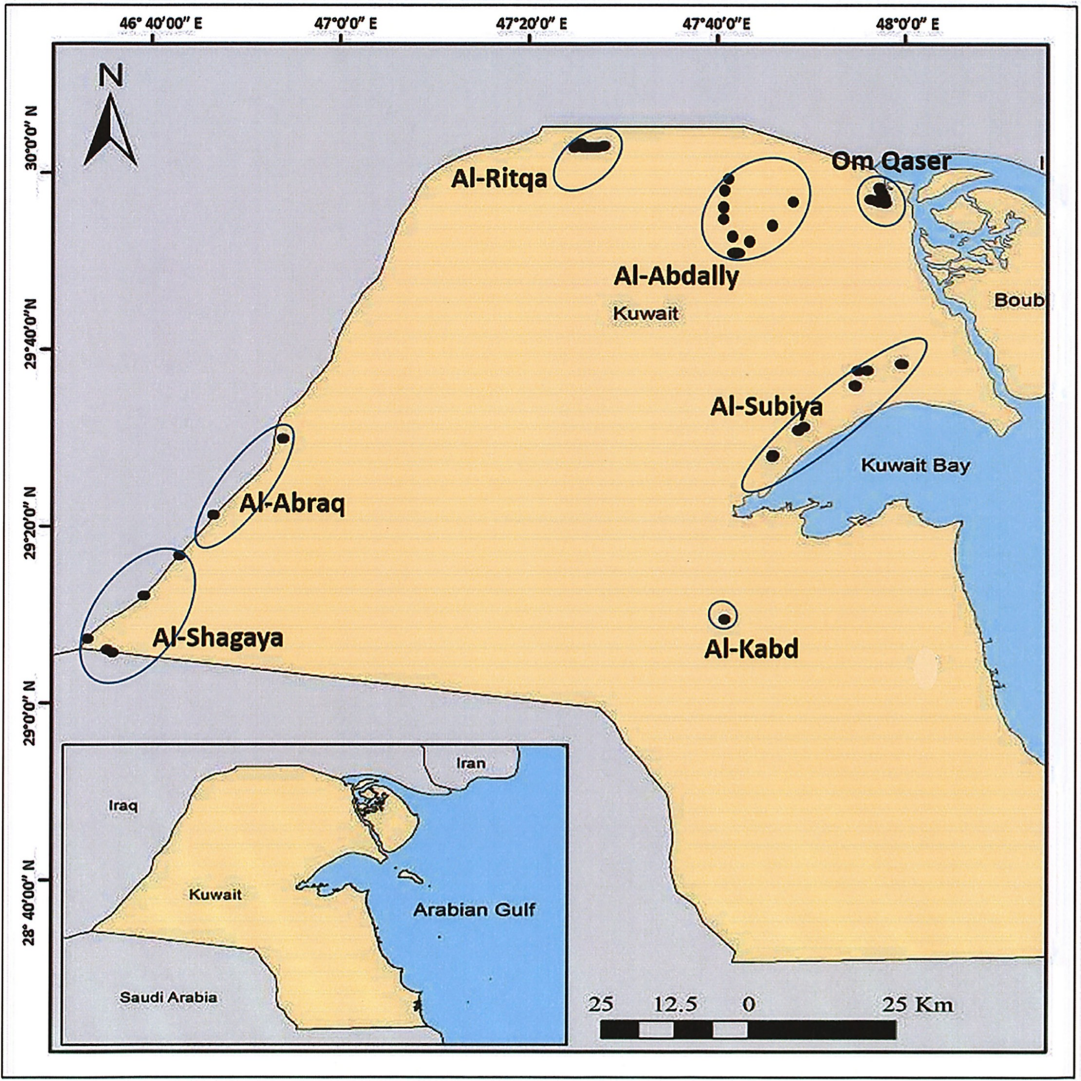
Locations where *H*. *salicornicum* populations were sampled in Kuwait. Locations were plotted on the Kuwaiti map based on the GPS coordinates for each specimen using the ArcMap software v 10.4.1.

Kuwait is a semi-arid country with the smallest area of 17,818 km^2^. Located in the north eastern corner of the Arabian peninsula, it shares border with Iraq and Saudi Arabia. A survey was conducted to locate the populations of *H*. *salicornicum* through direct searching as the species is retreating from Kuwait. Consultations were done with botanical experts to pinpoint the habitats where healthy populations of the plants could be found. Six areas from the Central, Northern and Southern part of Kuwait such as Al-Subiya, Om-Qaser, Al-Ritqa, Al-Shagaya, Al-Abraq and Al-Abdally, were identified for collection (Fig 1). Field visits were done in the designated areas and special permissions (throuh the Ministry of Interior, Kuwait) availed to enter restricted areas such as Al-Shagaya and Al-Abraq. Samples of Rimth were collected from these locations in Kuwait from January to June, 2016–2017. A total of 108 samples were collected from plants that were at least 30 m apart, depending upon its availability (Table 1). A single specimen of *H*. *salicornicum* was found in Al-Kabd area, the location of which was marked on the map, however, it was not included in the ISSR analysis. GPS coordinates were recorded for each plant sample (S1 Table), and plotted on the Kuwaiti map using the ArcMap software v 10.4.1 (Esri, Redlands, CA). Young leaf samples and shoots were collected and immediately placed in polythene bags. They were transported on ice to the lab. Samples were appropriately labeled and kept at -80°C until further use.

**Table 1:** Population, area and number of samples collected for *H*. *salicornicum*.

S. No #	Plant	Locations	Area of site of collection (km^2^)	No.of Samples collected
1	*Haloxylon salicornicum*	Al-Subiya	60.0	20
2		Om Qaser	19.9	20
3		Al-Ritqa	19.7	20
4		Al-Shagaya	49.3	25
5		Al-Abraq	14.0	10
6		Al-Abdally	99.8	13
	**Total**	**6**		**108**

### DNA extraction

DNA isolation from leaf tissues was carried out using GenElute Plant Genomic DNA Miniprep Kit (Sigma, St. Louis, MO), as per the manufacturer’s instructions. One hundred mg of leaf tissue was weighed and ground to a fine powder in liquid nitrogen using a pre-chilled mortar and pestle. Cell lysis followed by protein precipitation and washing was carried out using the different reagents supplied in the kit. DNA purity (Absorbance ratio A260/A280) and quantity (Absorbance at 260 nm) were measured by the Nanodrop (Thermo Scientific, Carlsbad, CA) and Qubit fluorometer (Thermo Fisher Scientific, Carlsbad, CA). All the samples of *H*. *salicornicum* were run on 0.8% of agarose gel to check the intactness of the isolated DNA. The samples were normalized at 10 ng/μl for ISSR analysis following the Qubit concentrations.

### PCR amplification

In the current study a total of 31 ISSR primers were screened with two random DNA samples initially. Sixteen primers that produced >5 polymorphic bands were chosen for subsequent analysis post optimization for the best annealing temperature (S2 Table). PCR was carried out with the 5x HOT FIREPol Blend Master Mix (Solis Biodyne, Estonia). A total reaction volume of 20 μl was prepared by adding 4 μl of master mix containing 5X buffer, 200-μM dNTPs, 1.5-mM MgCl_2_ and HOT FIREPol DNA polymerase. Primers (2 μl) were added at a concentration of 10 μM to yield a final concentration of 1.5 μM in the final mix. Ten nanograms of DNA per microlitre was added to the PCR reaction mix. Reaction was carried out in Veriti Thermal Cyclers (Applied Biosystems, Grand Island, NY) at specific annealing temperatures depending on the melting temperature (Tm) of each primer. The steps for PCR reaction were, initial enzyme activation at 95°C for 12 min followed by 45 cycles of denaturation at 95°C for 45 s, annealing at 45–60°C for 45 s and extension at 72°C for 1.5 mins. Final extension step was carried out for 7 min at 72°C. DNA bands were visualized on 1.8% agarose gels (13 cm long and 15 cm wide) ran at 5V/cm for 2 h. Gel images were documented using the gel documentation system (Chemidoc MP, BioRad, USA). One Kb and 100 bp ladder were used as reference. ISSR primers that produced polymorphic bands were repeated twice independently with three technical replicates for each sample (Gel replicability score ~ 93%; S3 Table). All the technical replicates produced banding patterns resembling each other as observed on the agarose gels (S1 Fig).

### Data scoring and analysis

All the samples were amplified and produced clear, reproducible bands (S2 Fig) which were scored as present (1) or absent (0), using the BioNumerics (v7.5; Applied Maths, Belgium) software. A binary matrix (1/0) was generated containing 195 loci which was used for subsequent data analysis for diversity and population structure (S4 Table).

The informativeness of ISSR markers was evaluated using the polymorphic information content (PIC) resolving power (RP), mean resolving power (MRP), marker index (MI) and Shannon’s index (H′). PIC is the probability in detecting polymorphism by a primer PIC  =  1-Σ(pi)^2^, where Pi is the frequency of the ith allele [27]; RP is the ability of each primer to detect level of variation between individuals and was calculated according to Prevost and Wilkinson [28]: RP  =  ΣIb where Ib (band informativeness) takes the values of 1–[2|0.5–p|], where p is the proportion of individuals containing the band. MI for each primer was calculated as a product of the polymorphic information content and effective multiplex ratio (MI  =  PIC × EMR [29]. Shannon’s index (H′) was calculated by the formula H′ = -pilnpi [30].

Genetic diversity within each population was estimated through percentage of polymorphic loci (P), mean effective number of alleles (*N*e), mean expected heterozygosity (*H*_E_), mean Shannon’s Information Index (*I*), Nei’s gene diversity (*h'*), and Nei’s pairwise genetic distances by using the GenAlEx 6.5 software [31,32]. The POPGene v 1.32 [33] software was used to calculate the overall Genetic differentiation (G_ST_ = H_T_-H_S_/H_T_). The G_ST_ was corrected according to Hedrick [34] and Jost [35] to obtain the estimates for G’_ST_ and D, respectively. We also performed an analysis of molecular variance (AMOVA) to examine the distribution of genetic variability within and among populations using the Arlequin software (version 3.5) [36] and estimated an overall F_ST_ (Fixation index). Based on the F_ST_ the Gene flow (N_M_ = 1(1/F_ST_-1)/4 was also calculated [37]. The pairwise F_ST_ from the Arlequin software were used to derive the Slatkins relationship (F_ST_/1-F_ST_) to create an isolation by distance plot through the Mantel’s test employing 10,000 permutations [36].

Population structure was determined by the neighbour-net split decomposition network generated by SPLITS Tree v4.6 and bootstrapping runs of 1000 replicates [38], a PCoA analysis of pairwise F_ST_ between the populations [36] and Bayesian model-based clustering analysis using the software program STRUCTURE 2.3.3 [39]. The admixture model and correlated allele frequencies were used for each run with a burn-in period of 1,000 and 100,000 Markov chain Monte Carlo replications. The optimal K value, which indicates the number of genetically distinct clusters in the data, was determined from 10 replicate runs for each value of K [40]. The value of ΔK was based on the change in the log probability of the data between successive K values. Structure Harvester version 6.0 [41] was used to calculate parameters described by Evanno et al. [40]. The usepopinfo option of STRUCTURE was employed after K was determined by the Evanno’s method to estimate Q (proportion of membership of each pre-defined population in each of the clusters).

## Results

### ISSR polymorphism

For *H*. *salicornicum*, 16 primers produced 195 unambiguous and reproducible bands. Out of them167 were polymorphic (86.5%). Smeared and faint bands were not considered for analysis. The bands ranged from 135–1729 bp. Number of bands per primer ranged from minimum 8 (ISSR 8, 13, and 809) to maximum 21 (ISSR 826) with an average count of 10.5 bands per primer. All the primers had a high discriminating power as observed by their PIC (mean-0.31) values. Highest PIC was obtained with ISSR 7 (0.46) and the lowest (0.18) with ISSR 13 and 23 (Table 2). Other parameters such as MI, RP, MRP, and H' were also recorded for all the primers. Average RP of 3.76 (Max- 6.6, ISSR 5; Min-2.2, ISSR 809), MRP of 0.36 (Max-0.49, ISSR 3; Min-0.20 ISSR 7), MI of 3.39 (Max 8.0, ISSR 826; Min 1.4, ISSR 13), and H' of 0.326 (Max 0.504, ISSR 826; Min 0.221, ISSR 8) were observed. There was a positive correlation between the MI and PIC values (*r*^2^ = 0.813, P<0.05) and PIC and RP (*r*^2^ = 0.615, P<0.05).

**Table 2:** ISSR primer core sequences, number of bands and polymorphism content for *H*. *salicornicum*.

Primer Code	Primer Sequence	SB	PB	PPB	BR	PIC	RP	MRP	MI	H'
ISSR 7	(AGC)_6_GA	14	12	85.7	233–1237	0.46	2.3	0.20	5.5	0.290
ISSR 3	(GA)_8_TC	11	10	90.9	250–1051	0.45	4.9	0.49	4.5	0.266
ISSR 826	(AC)_8_AG	21	19	90.5	322–1522	0.42	6.6	0.35	8.0	0.504
ISSR 5	(AAG)_6_GC	15	10	66.7	168–1729	0.40	6.7	0.67	4.0	0.347
ISSR 10	CCCGGATCC(GA)_8_	10	10	100.0	344–1027	0.38	4.1	0.41	3.8	0.262
ISSR 2	CA(GA)_7_GT	16	14	87.5	155–1137	0.37	6.5	0.46	5.2	0.424
ISSR 8	(GGC)_6_TA	8	8	100.0	650–1557	0.34	3.2	0.40	2.7	0.221
ISSR 810	(GA)_8_T	10	8	80.0	251–1006	0.34	2.8	0.35	2.7	0.260
ISSR 820	(AC)_9_C	11	10	90.9	269–920	0.34	4.3	0.43	3.4	0.300
ISSR 17	GATCCA(GCA)_6_C	13	13	100.0	135–1649	0.26	3.9	0.30	3.4	0.376
ISSR 809	(AG)_10_G	8	7	87.5	408–1327	0.25	2.2	0.32	1.8	0.233
ISSR 11	CCCGGATCC(CT)_8_	9	8	88.9	516–1516	0.23	2.4	0.31	1.8	0.263
ISSR 18	GAT(CAT)_8_C	12	9	75.0	626–1386	0.22	3.1	0.35	2.0	0.352
ISSR 12	CCCGGATCC(GT)_8_	12	12	100	497–1424	0.20	2.8	0.24	2.4	0.360
ISSR 13	CCCGGATCC(CA)_8_	10	8	80.0	424–1332	0.18	1.7	0.22	1.4	0.300
ISSR 23	(GA)_11_A	15	9	60.0	271–1031	0.18	2.5	0.28	1.6	0.445
**Total**		**195**	**167**		**135–1729**					
**Average**			**10.4**	**86.5**		**0.31**	**3.76**	**0.36**	**3.39**	**0.325**

SB- No. of scored bands, PB- No. of polymorphic bands, PPB- percentage of polymorphic bands, BR- Band range, PIC-polymorphic information content, RP- Resolving power, MRP- Mean resolving power, MI-Marker Index, H'- Shannon’s diversity index

### Genetic diversity

In *H*. *salicornicum*, an average number of observed alleles (*N*_*a*_*)* ranged from 1.144 (Al-Abdally) to 1.610 (Al-Subiya). Relatively higher levels of allelic diversity (*N*_*e*_) were observed in the populations of Al-Subiya, Om Qaser and Al-Ritqa, while moderate levels were recorded in Al-Shagaya and Al-Abraq and a lower level in Al-Abdally (Table 3). Similar pattern was observed for Shannon’ diversity index (*I)* and Nei’s gene diversity *(h')* with highest values in Al-Subiya (*h'*-0.254; *I*-0.375) and lowest in Al-Abdally (*h'*-0.101; *I*-0.0.138). Al-Shagaya and Al-Ritqa showed *I* and *h'* values comparable to Al-Subiya and Om Qaser. The overall average values of *h'* and *I* were 0.200 and 0.303 respectively. The percentage values of polymorphic loci were ≥ 60% for Al-Subiya, Om Qaser, and Al-Ritqa; ≥ 45% for Al-Shagaya, and ≤ 27% in Al-Abraq with an average polymorphism above 50% in six populations of *H*. *salicornicum*. The populations within Al-Subiya, Om Qaser, Al-Ritqa, and Al-Shagaya showed relatively high values of *H*_*e*_ and *uH*_*e*_ (> 0.200) as compared to Al-Abraq and Al-Abdally (< 0.200). The genetic diversity was highest in the populations of Al-Subiya, followed by Om Qaser, Al-Ritqa, Al-Shagaya, Al-Abraq, and Al-Abdally.

**Table 3:** Measures of genetic diversity within the 6 populations of *H*. *salicornicum*.

Pop	*N*_*a*_	*N*_*e*_	*I*	*H*_*e*_	*uH*_*e*_	*h'*	%P
Al-Subiya	1.610	1.456	0.375	0.258	0.264	0.254	63.59%
Om Qaser	1.610	1.429	0.357	0.244	0.250	0.246	62.56%
Al-Ritqa	1.590	1.427	0.353	0.242	0.248	0.241	61.54%
Al-Shagaya	1.518	1.373	0.319	0.216	0.220	0.215	56.92%
Al-Abraq	1.431	1.337	0.276	0.189	0.196	0.144	48.21%
Al-Abdally	1.144	1.158	0.138	0.092	0.097	0.101	26.15%
**Mean**	**1.484**	**1.363**	**0.303**	**0.207**	**0.213**	**0.200**	**53.16%**

*N*_*a*_-Mean observed number of alleles; *N*_*e*_ -Mean effective number of alleles, *I-* Shannon’s diversity index, *He-* Expected heterozygosity; *uHe*- unbiased expected heterozygosity; *h'-*Nie’s gene diversity; %P- percentage of polymorphic loci

### Genetic differentiation

The Analysis of molecular variance (AMOVA) test was applied to the binary matrix data file to obtain information on the variation within and among populations by the Arlequin software. The results of the AMOVA indicated that most (77.83%) genetic variation was within populations, with a lesser variation (22.17%) among the populations (Table 4). the Fixation index was high enough (F_ST_ = 0.221, P = 0.000) in *H*. *salicornicum* to prevent inbreeding.

**Table 4:** Summary of the AMOVA results for 108 specimens of *H*. *salicornicum*.

Source	df	SS	Est. Var.	%	F_ST_	P(rand > = data)
**Among Pops**	5	501.216	4.271	22.2		
**Within Pops**	102	1727.218	16.575	77.8		
**Total**	**107**	**2191.843**	**21.296**	**100%**	**0.221**	**0.000**

d.f., degree of freedom; SS, sum of squared observations; Est. var. Estimated Variance; %- Percentage of variation; F_ST_. Fixation index obtained by applying F statistics over all loci. Significance test (P>0.05) was performed with 10100 permutations (over 20000 bootstraps)

The pairwise Nei’s genetic distances (*D*_S_) among populations were low ranging from 0.047 between Al-Ritqa and Al-Shagaya to 0.187 between Al-Abdally and Al-Subiya populations of *H*. *salicornicum* (Table 5). The pairwise F_ST_ were 0.094 between Al-Ritqa and Om Qaser to 0.447 between Al-Abdally and Al-Abraq.

Values of genetic differentiation G_ST_, G’_ST_ and D were estimated as 0.262, 0.327 and 0.335 respectively. Average gene flow (N_M_) based on the fixation index (F_ST_) among the populations was ~ 1 (0.88).

**Table 5:** Values of pairwise genetic distance (D, below the diagonal) and fixation index (F_ST_ above the diagonal) for six *H*. *salicornicum* populations in Kuwait.

	Al-Subiya	Om Qaser	Al-Ritqa	Al-Shagaya	Al-Abraq	Al-Abdally
**Al-Subiya**	***	0.130	0.165	0.194	0.270	0.390
**Om Qaser**	0.066	***	0.094	0.135	0.238	0.365
**Al-Ritqa**	0.076	0.052	***	0.113	0.236	0.365
**Al-Shagaya**	0.079	0.058	0.047	***	0.138	0.352
**Al-Abraq**	0.114	0.103	0.093	0.050	***	0.447
**Al-Abdally**	0.187	0.173	0.162	0.129	0.092	***

The p value corresponding to each pairwise F_ST_ was > = 0.00

### Clustering and population structure

Clustering analysis was performed to study the population structure of *H*. *salicornicum*. The approaches used were the split decomposition analysis, the Bayesian clustering and the principal coordinate analysis (PCoA).

**Fig 2:**
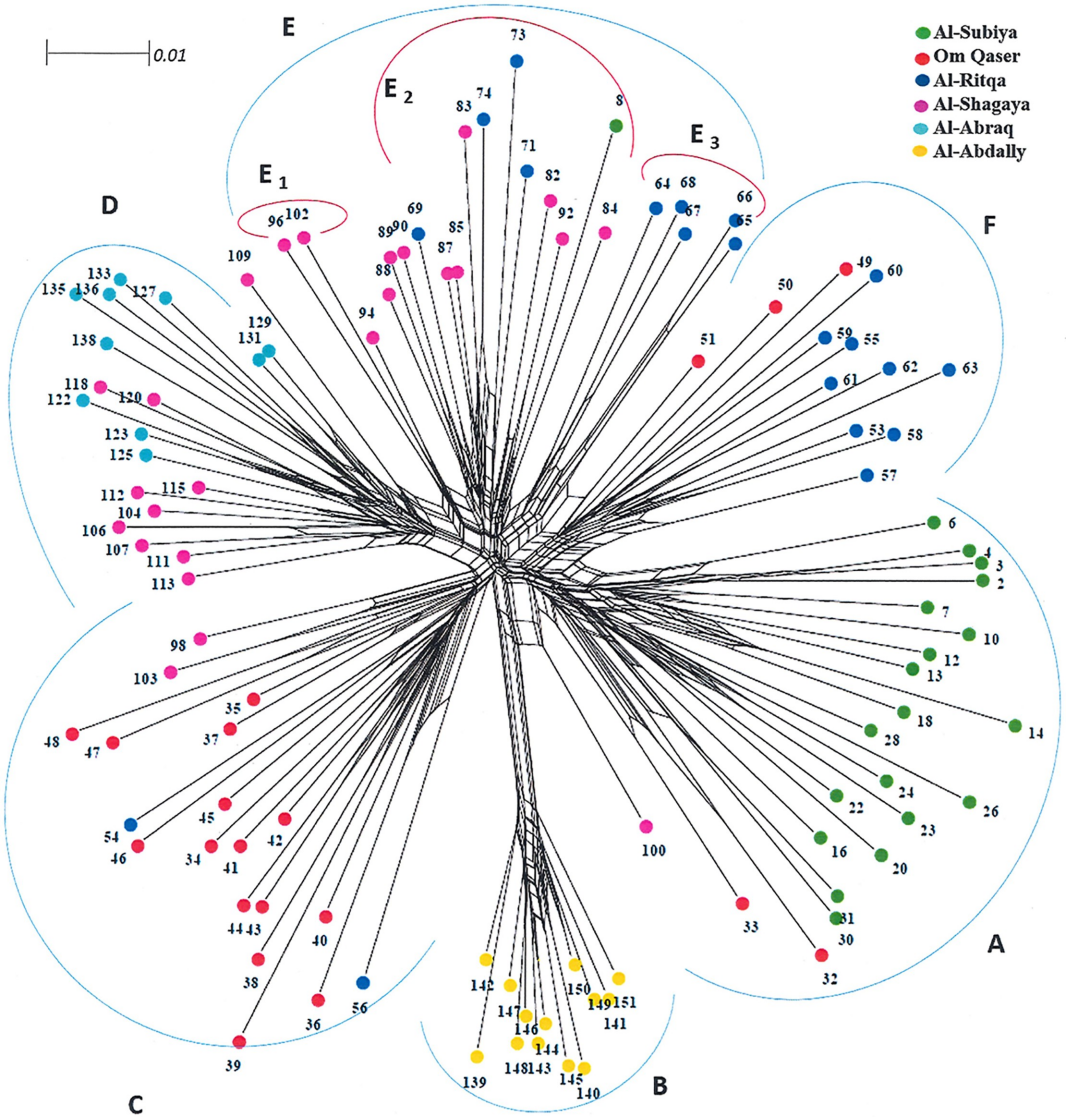
The splits tree dendogram demonstrating the genetic relatedness among the six *H*. *salicornicum* populations in Kuwait. Groups (A-F) showing extensive reticulation in consistency with high intrapopulation diversity.

The neighbour-net based split decomposition returned a Split tree dividing the *H*. *salicorncum* populations into six weakly separate sub-populations (Fig 2). Group-A in the Split tree consisted of Al Subiya populations with two samples from Om Qaser. Al-Abdally formed a distinct collection farest from the rest with no overlapping specimens from any of the populations in Group-B. Group C was Om Qaser with samples from Al-Shagaya and Al Ritqa. Group D mainly comprised all the populations of Al Abraq and half of the specimens from Al Shagaya. The remaining half of Al Shagaya clustered with Al Ritqa in Group E. A closer look at Group E revealed it could be further distributed into three subgroups (E1-E3). The last group was F with remaining populations of Al Ritqa and Om Qaser. Bootstrapping run with 1000 replicates yielded 88% confidence.

**Fig 3:**
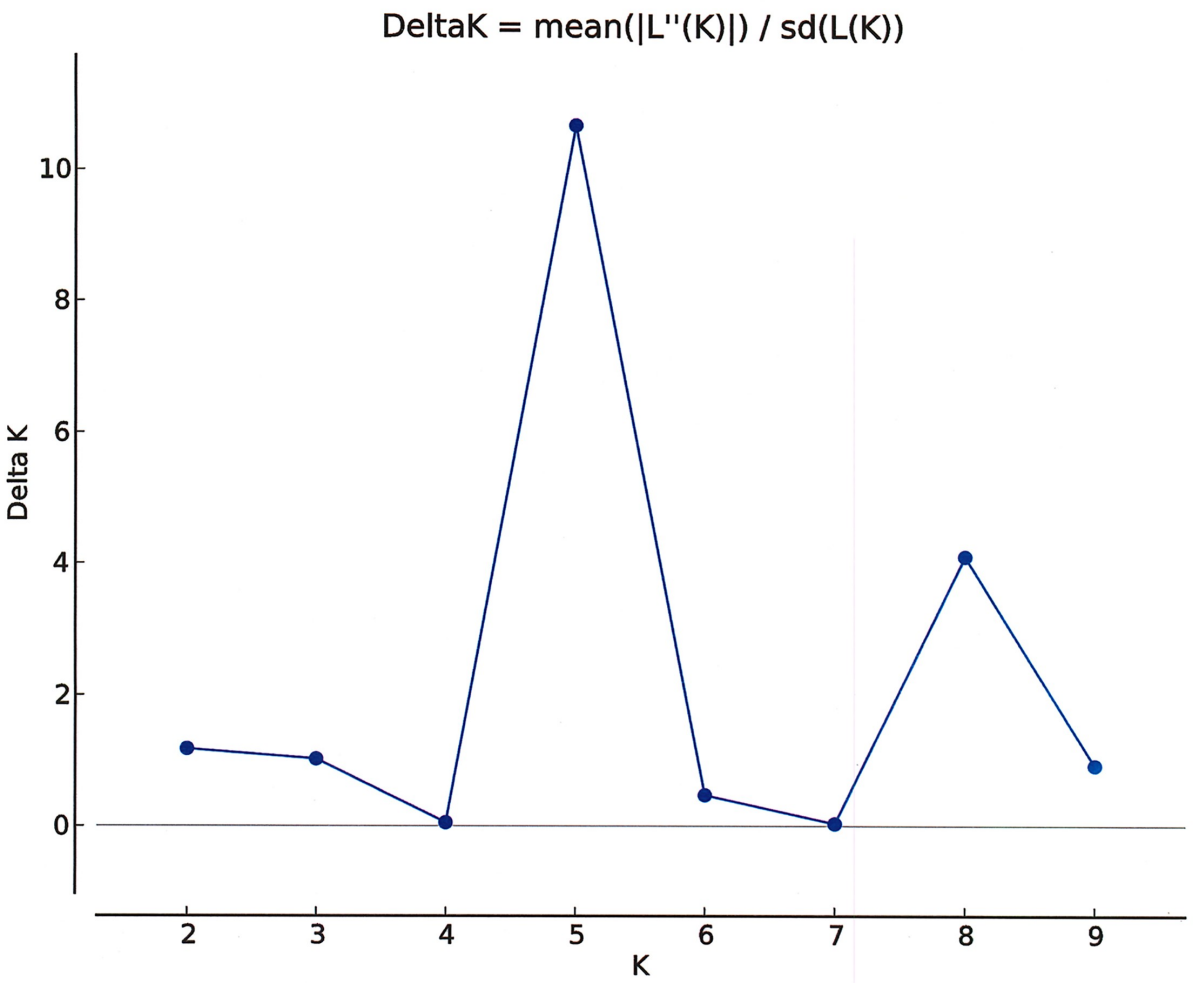
Relationship between ΔK and K as revealed by Structure Harvester. Estimation of the number of subgroups for the K values ranging from 1 to 10, by delta K (ΔK) values.

**Fig 4:**
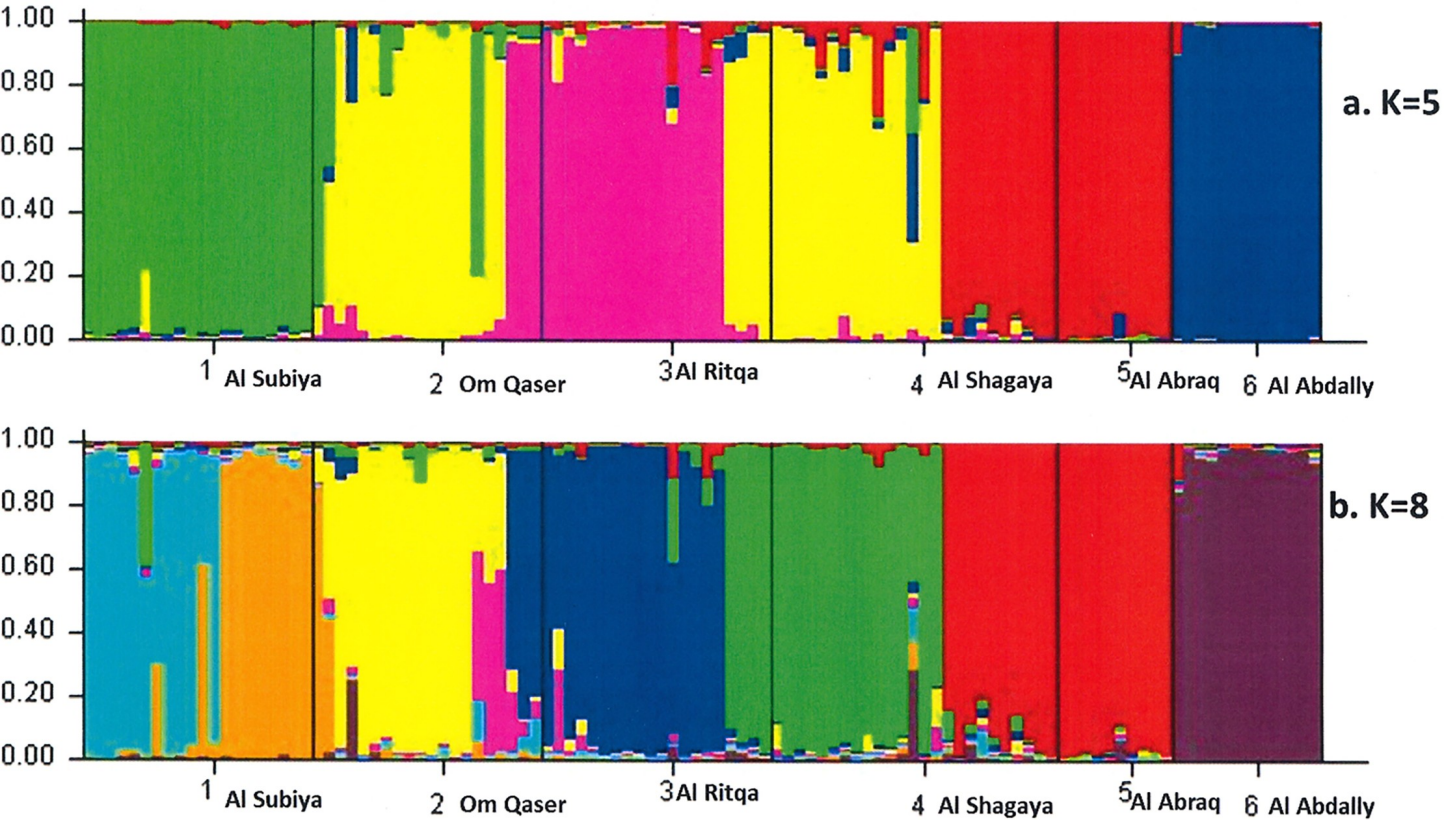
Bayesian model clustering of *H*. *salicornicum* by STRUCTURE. Numbers 1–6 represent one population (1-Subiya, 2-Om Qaser, 3-Ritqa, 4- Shagaya, 5-Abraq and 6-Abdally). Each colour represents one group. Number of groups (K) was set to 5 (a) and 8 (b) based on the Evanno’s method. The length of coloured segment denotes the proportion of each population in each group.

To estimate the reliability of the likely cluster groups analyzed already, the genetic structure of the 6 populations was calculated using the STRUCTURE software. The number of genetic groups (*K*) showed a visible peak at 5 and a smaller peak at 8 (Fig 3) by Evanno’s method, indicating that 5 groups should be distributed across all the *Haloxylon* populations (Fig 4A). A detailed study of the clusters revealed that group-1 (green) contained the populations of Al Subiya (Q = 0.994) with trivial proportions of Om Qaser (Q = 0.013) and Al Shagaya (Q = 0.014), group II (yellow) was the biggest with larger populations of Om Qaser (Q = 0.846) and a small fraction of Al Shagaya (Q = 0.019), Group III (pink) had the major populations of Al Ritqa (Q = 0.953) overlapping with Om Qaser (Q = 0.135), Group IV (red) comprised of Al Shagaya (Q = 0.962) and Al Abraq (Q = 1.0) whereas the last group V (blue) had distinctly the populations of Al Abdally (Q = 0.991) populations. Another structure analysis was performed pertaining to an additional peak obtained at K = 8 through the Evanno’s method. The populations of Al Subiya and Om Qaser were distributed into four groups represented by different colors in Fig 4B. These results were in partial agreement with Splits tree diagram where group E could be subdivided into 3 sub clusters, yielding a total of 8 groups.

**Fig 5:**
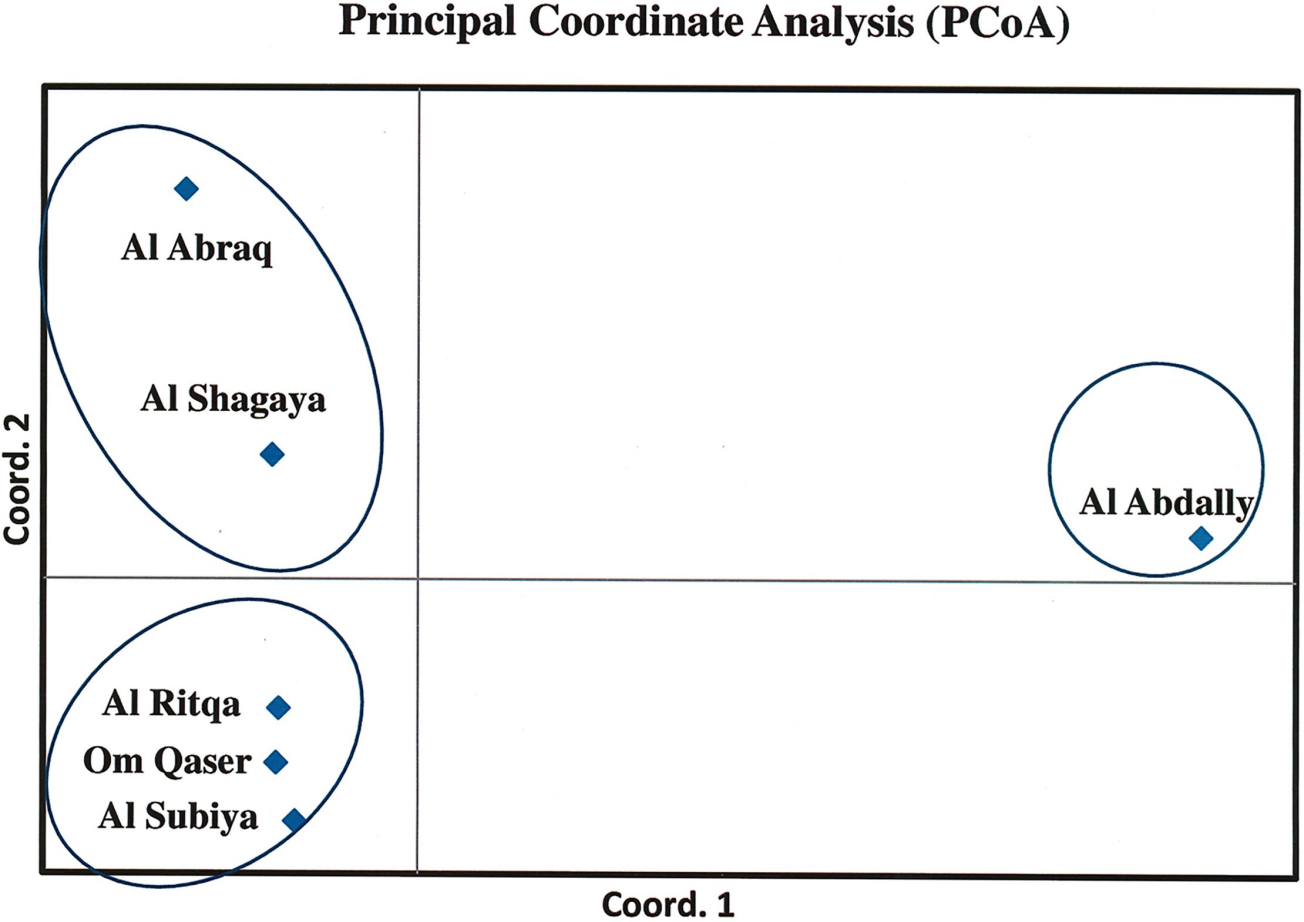
Scatter plot obtained from principal coordinate analysis of the pairwise F_ST_ matrix of six populations of *H*. *salicornicum* derived from 195 polymorphic microsatellite (ISSR) loci.

The percentage of variation along the first three axes in PCoA were, 43.61%, 26.61% and 15.08%. The PCoA clustered the six populations into three groups. Al Abdally formed a distinct cluster, however the populations of Om Qaser Al Ritqa and Al Subiya combined as one collection, whereas Al Shagaya and Al Abraq grouped together (Fig 5).

**Fig 6:**
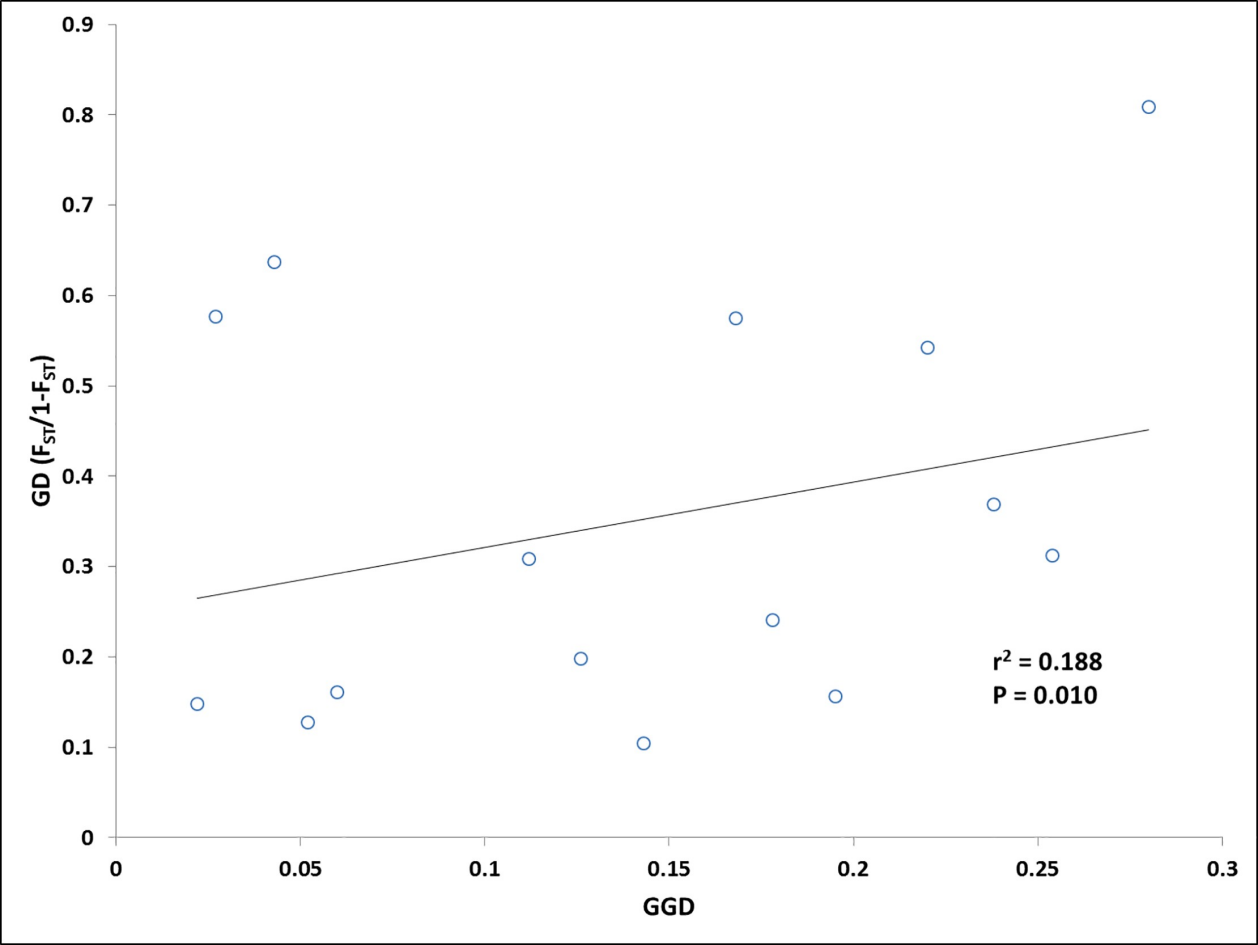
IBD plots of the genetic distance and geographical distance. The Y axis represents the genetic distance (F_ST_/1-F_ST_), and the X axis represents the geographical distance.

The isolation by distance analysis carried out through the Mantel’s correlation test demonstrated that *H*. *salicornicum* witnessed a weak correlation (*r*^*2*^- 0.188; P = 0.013) among the genetic and geographic distance (Fig 6). The significance at a confidence level of 95% (P≤0.05) was tested with 10,000 permutations.

## Discussion

This study provides a first report on genetic variation and population structure of *H*. *salicornicum* distributed in Kuwait. Under the changing climatic conditions our findings will be crucial for effective management and developing conservation strategies for this native plant.

### ISSR polymorphism

To understand the extent of genetic diversity, genetic structure and differentiation among *H*. *salicornicum* populations occurring in different geographic regions of Kuwait, the PCR-based ISSR markers, were selected based on their applicability in desert plants that were used for both inter- and intra-population analysis [20–23]. The markers were able to detect 86.5% of polymorphism in the current study. This level of polymorphism is suggestive of considerable levels of genetic variation in the populations of *H*. *salicornicum* grown in Kuwait. A higher percentage of polymorphic loci of 96% were recorded in the closely related *H*. *ammodendron* from China [42] and 98.5% in *H*. *salicornicum* distributed in the Indian Thar desert [23]. The high rate of polymorphism in *Haloxylon* communities would be attributed to its demographic features. Kuwait being an arid land with increased level of soil salinity and temperature fluctuations provides a stressful atmosphere for plant growth [42]. In past several studies have demonstrated a correlation between higher degrees of polymorphism as well as the level of environmental stress and heterogeneity [43–45]. The high discriminating power of 0.31 also indicates a balanced distribution of allele frequencies in *H*. *salicornicum* communities of Kuwait. In comparison to present study, the few other studies of population genetic diversity of long-lived desert perennials also revealed high levels of genetic polymorphism [24–25]. A significant factor in the stability of desert woody plants is their intraspecific polymorphism [46].

### Genetic diversity

The level of genetic diversity is often correlated with its range of distribution. In the present study, *H*. *salicornicum* is narrowly distributed within an area of 17,818 km^2^. We hypothesized that the genetic diversities of *H*. *salicornicum* populations would be low due to small sample size distributed in a small area. The values of *uHe* observed (0.213) under the current study was approximately twice of that of the rare plants. According to Cole’s observations, *He* (expected heterozygosity) for a rare plant was recorded as 0.113, whereas, for a common plant, it was 0.150 [47]. The number of effective alleles in *Haloxylon* populations refers to the occurrence of rare microsatellite alleles imparting much of the diversity in the species. Higher levels of expected observed and expected heterozygosities were recorded in *H*. *aphyllum* [46].

The level of heterozygosities obtained are indicative of more genetic variation and thus leaving behind a scope for population expansion. Consequently higher diversity indices (*I* = 0.330; *h’* 0.200) among the *Haloxylon* populations were observed in our study. Similar findings were reported for *Haloxylon* distributed in China [21], India [22] and Saudi Arabia [48, 49]. This could be a result of many factors such as breeding system, seed dispersal, genetic drift, evolutionary history as well as life form. In the present scenario the plant propagates naturally through seeds dispersed by winds, run-off water and insects [50]. The seeds are viable upto 1 year [51]. Germination is severely inhibited above 30°C [52] and an increase in salinity [53]. The flowering season is between September and October. Mature fruits are produced during December- January. [7] The seeds of *H*. *salicornicum* are small grayish in color and attached to a perianth, which gives a wing-like appearance assisting in their long distance dispersal by wind [52,53]. The above mentioned factors contribute to the high genetic diversity in *Haloxylon*.

### Gene differentiation and gene flow

In order to study the accumulation of differences in the allelic frequencies, partitioning of genetic differentiation were recorded in the current investigation. Reproductive biology is considered as the driving force behind the population differentiation [54]. Generally AMOVA partitioning demonstrates 10–20% of the genetic variation to be found among the populations in an out-crossing species, whereas for a selfing species this reaches upto 50%. *Haloxylon salicornicum* being an cross pollinated [50] species followed an analogous pattern of genetic differentiation. Our findings were in agreement with related species of *Haloxylon* distributed in other Asian countries [23, 48,49,55]. The proportion of variation within populations was as high as 89.4% whereas among population was approximately 10% as detected by eight ISSR markers on 195 scorable loci of *H*. *ammodendron* species in China [54]. Similar values (79% variation within and 21% among populations) were also obtained through different marker system of RAPD and ISJ which amplified 67 loci in *H*. *salicornicum* collected from six locations of Jaisalmer district in India [23]. The genetic variability of *Haloxylon* species revealed in this study is important with respect to the ecological conservation and restoration practice. A major proportion of the variability present within populations suggests that smaller numbers of populations will be required for effective conservation whereas, the high genetic homogeneity among populations of suggests that the exact population used as a source of material for restoration may be less important in this species.

Relative to the low genetic diversity among the populations the overall fixation index was high enough to prevent allele fixation or inbreeding. For plant populations values of F_ST_ greater than 15% fall under significant categories [56, 57]. This was in agreement with its closest analogue -G_ST_ (low -<0.15; medium—0.05–0.15; or high- >0.15). In addition, corrected G’_ST_ and Jost’s D between 0 to 1 further supported the fact that the *Haloxylon* populations under study were not completely homozygous and considerably differentiated genetically [34,35,37]. Comparable level of genetic differentiation have also been reported in closely related *H*. *ammodendron* in China [42,54].

Genetic differentiation is inversely proportional to the gene flow. Slatkin [58] posited N_M_ > 1 as an indicator of sound gene flow necessary to maintain genetic variation when population size is small. Overall gene flow ≤ 1 (0.880) was recorded in the present study. An increased geneflow is suggestive of decreased inbreeding. The gene flow (N_M_) observed in the present investigation less than 1 is deemed to be sufficient to maintain genetic variation among the Rimth populations. Comparable levels of gene flow were also recorded in *H*. *ammodendron* in China [21].

### Population structure

The correlation between the genetic and geographical distance (Mantels test r = 0.188 p< 0.05) revealed that the populations of *Haloxylon* in Kuwait were weakly isolated by distance. The clustering pattern revealed that the groupings were largely in agreement with the geographical distribution of the accessions. Al Abraq always clustered with Al Shagaya owing to their geographic proximity. The populations of Om Qaser, Al Ritqa and Al Shagaya formed overlapping clusters with each other in the Splits tree and the Structure Plot. This could be explained by the admixture events detected from the sampling of hybrid individuals as subpopulations come into contact via dispersal or from sharing a border [59]. The pairwise F_ST_ values were also recorded to be relatively lower among the populations in close vicinity. The PCoA was suggestive of interbreeding between the specimens of Al Abraq and Al Shagaya as well as the populations of Al Subiya, Al Ritqa and Om Qaser. Al Abdally was the farest and most distinct group in all the cases. This would be attributed to, an unknown sympatric speciation event occurring in the area restricting the gene flow with neighboring populations. However, more information on phenotypic variations needed to be gained for species in Al Abdally region.

We observed that the individual clusters were not far away from each other. The weak site differentiation among the clusters can be explained due to the low genetic distance among the populations [60]. From an evolutionary perspective, this indicates mixed ancestry of *Haloxylon* in Kuwait. Another explanation given for the weak population structure is the effectively maintained geneflow. In absence of any topographical barriers the genes among the populations effectively communicate with each other. In the present case the number of migrants among the populations almost equaled to 1 (N_M_ = 0.880). Migration is a common mechanism that erodes population structure [61]. The absence of relevant barriers resulted in low geographic and genetic distances and therefore facilitating the dispersal of seeds among the populations. Therefore we conclude that a weak population structure of *Haloxylon* species is observed in Kuwait. Sewell Wright [62] described that pairs of populations geographically close to each other will be more genetically similar because their individual critters, or their seeds, or pollen, or larvae easily travel short distances.

### Conservation implications

From a conservation standpoint, the high genetic diversity maintained within *H*. *salicornicum* and the significant genetic differentiation within its populations are encouraging because these characteristics are advantageous for preserving genetic resources and broadening the genetic basis of these native plants. However, we detected a recent bottleneck event occurring in population structure, suggesting that individual populations may suffer from a dramatic decline in population size. Given the small number of existing populations and the low, genetic distances among the populations, these populations should be conserved as much as possible to maintain their current status, especially the populations with higher diversity, such as populations in Al-Subiya for *Haloxylon*, should be protected as a priority for *in situ* conservation.

## Supporting information

S1 Fig PCR with selected ISSR primers (23, 810 and 3) to check for reproducibility in *H. salicornicum*. Lanes 1–3 represent 3 technical replicates in first set of PCR (Day 1), Lanes 4–6 represent 3 technical replicates in second set of PCR (Day 2); M—Marker (100bp + 1Kb ladder).
(TIF)

S2 Fig PCR band profiles. Generated by ISSR primer 2 in *H*. *salicornicum* samples, Marker (100bp + 1Kb ladder).
(TIF)

S1 Table GPS coordinates of the collected specimens of *Haloxylon*. (DOCX)

S2 Table ISSR primers and their optimum annealing temperatures. (DOCX)

S3 Table Gel Replicability score for ISSR markers. (XLSX)

S4 Table Binary file of 108 samples of *H. salicornicum* collected from six locations of Kuwait. (XLSX)
