# Supplementary figures and images for: Genetic diversity and population structure of Haloxylon salicornicum moq. in Kuwait by ISSR markers
Source: PLoS One. 2018 Nov 21;13(11):e0207369. doi: 10.1371/journal.pone.0207369 (PMC6248962; doi:10.1371/journal.pone.0207369)

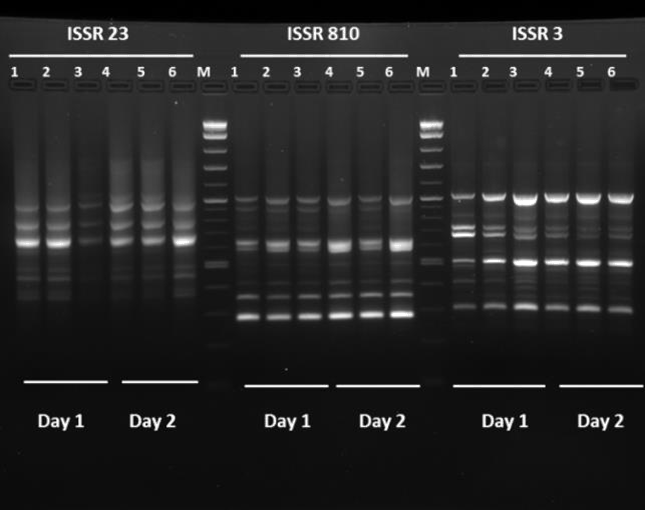

Supplement: S1 Fig — Lanes 1–3 represent 3 technical replicates in first set of PCR (Day 1), Lanes 4–6 represent 3 technical replicates in second set of PCR (Day 2); M—Marker (100bp + 1Kb ladder). (TIF) [file pone.0207369.s001.tif]

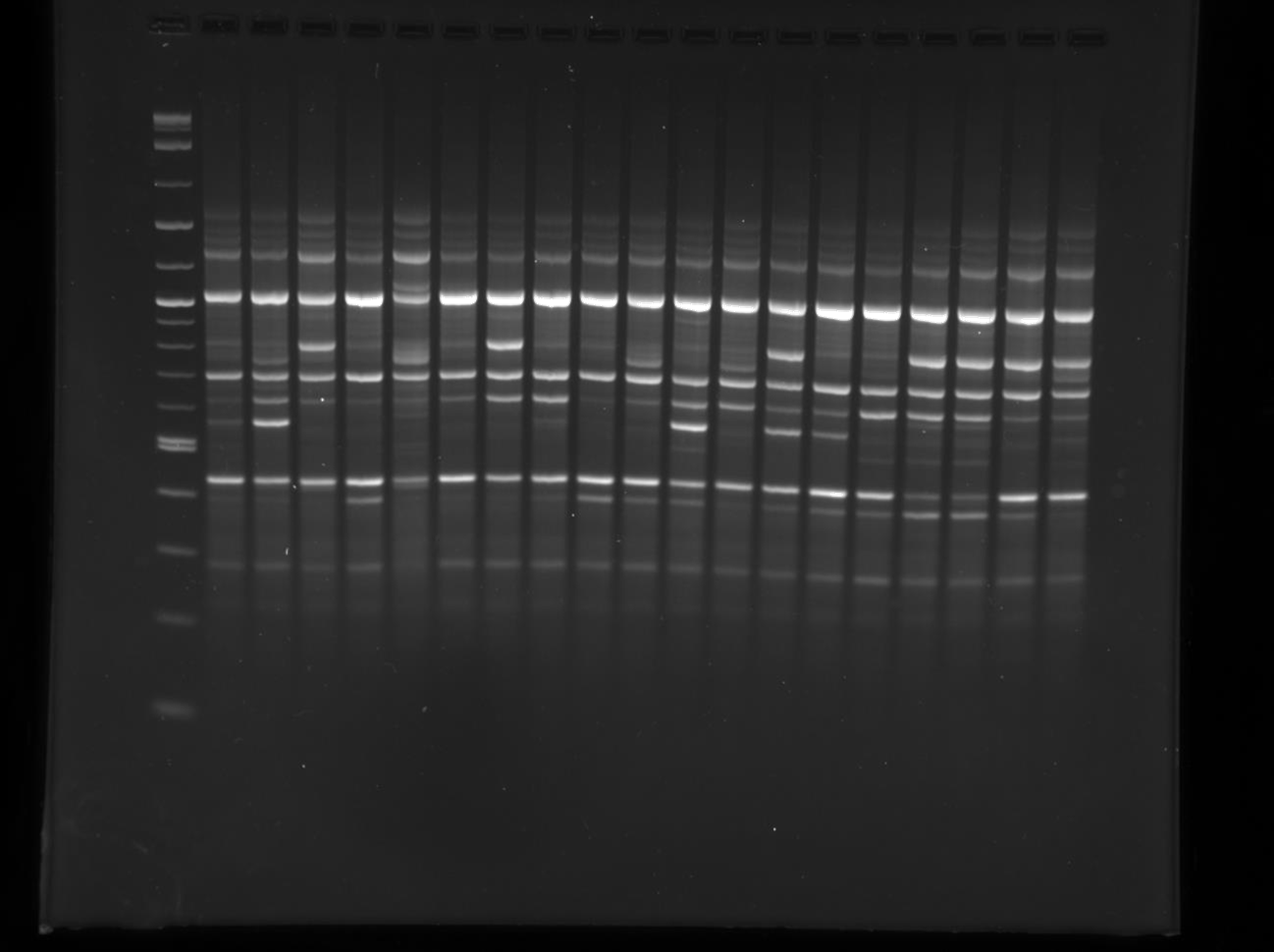

Supplement: S2 Fig — Generated by ISSR primer 2 in H. salicornicum samples, Marker (100bp + 1Kb ladder). (TIF) [file pone.0207369.s002.tif]
