# Supplementary material for: Genetic diversity and population structure of Haloxylon salicornicum moq. in Kuwait by ISSR markers
Source: PLoS One. 2018 Nov 21;13(11):e0207369. doi: 10.1371/journal.pone.0207369 (PMC6248962; doi:10.1371/journal.pone.0207369)
Supplement: S1 Table — (DOCX) [file pone.0207369.s003.docx]

| **S. No** | **Sample No.** | **GPS** | | |
| --- | --- | --- | --- | --- |
|  |  | **N-DM** | **E-DM** | **Area** |
| 1 | HS-Kb-001 | 29.64798 | 47.99595 | Al Kabd |
| 2 | HS-Sb-002 | 29.64785 | 47.99630 | Al Subiya |
| 3 | HS-Sb-003 | 29.64763 | 47.99667 | Al Subiya |
| 4 | HS-Sb-004 | 29.64767 | 47.99640 | Al Subiya |
| 5 | HS-Sb-006 | 29.64825 | 47.99508 | Al Subiya |
| 6 | HS-Sb-007 | 29.64842 | 47.99458 | Al Subiya |
| 7 | HS-Sb-008 | 29.64857 | 47.99398 | Al Subiya |
| 8 | HS-Sb-010 | 29.63592 | 47.93452 | Al Subiya |
| 9 | HS-Sb-012 | 29.63548 | 47.91730 | Al Subiya |
| 10 | HS-Sb-013 | 29.63485 | 47.91803 | Al Subiya |
| 11 | HS-Sb-014 | 29.60763 | 47.91375 | Al Subiya |
| 12 | HS-Sb-016 | 29.60727 | 47.91357 | Al Subiya |
| 13 | HS-Sb-018 | 29.60685 | 47.91352 | Al Subiya |
| 14 | HS-Sb-020 | 29.60630 | 47.91327 | Al Subiya |
| 15 | HS-Sb-022 | 29.52885 | 47.82292 | Al Subiya |
| 16 | HS-Sb-023 | 29.52245 | 47.81375 | Al Subiya |
| 17 | HS-Sb-024 | 29.52278 | 47.81307 | Al Subiya |
| 18 | HS-Sb-026 | 29.47587 | 47.76883 | Al Subiya |
| 19 | HS-Sb-028 | 29.47448 | 47.76812 | Al Subiya |
| 20 | HS-Sb-030 | 29.47243 | 47.76712 | Al Subiya |
| 21 | HS-Sb-031 | 29.95552 | 47.95023 | Al Subiya |
| 22 | HS-Oq-032 | 29.95483 | 47.95205 | Om Qaser |
| 23 | HS-Oq-033 | 29.95390 | 47.95395 | Om Qaser |
| 24 | HS-Oq-034 | 29.95317 | 47.95790 | Om Qaser |
| 25 | HS-Oq-035 | 29.95317 | 47.95790 | Om Qaser |
| 26 | HS-Oq-036 | 29.95268 | 47.96153 | Om Qaser |
| 27 | HS-Oq-037 | 29.95163 | 47.96648 | Om Qaser |
| 28 | HS-Oq-038 | 29.95465 | 47.96520 | Om Qaser |
| 29 | HS-Oq-039 | 29.95805 | 47.96425 | Om Qaser |
| 30 | HS-Oq-040 | 29.96125 | 47.96358 | Om Qaser |
| 31 | HS-Oq-041 | 29.96233 | 47.96362 | Om Qaser |
| 32 | HS-Oq-042 | 29.96957 | 47.96027 | Om Qaser |
| 33 | HS-Oq-043 | 29.97032 | 47.95972 | Om Qaser |
| 34 | HS-Oq-044 | 29.97567 | 47.95655 | Om Qaser |
| 35 | HS-Oq-045 | 29.98145 | 47.95492 | Om Qaser |
| 36 | HS-Oq-046 | 29.95200 | 47.96487 | Om Qaser |
| 37 | HS-Oq-047 | 29.95470 | 47.95242 | Om Qaser |

| 38 | HS-Oq-048 | 29.95523 | 47.95117 | Om Qaser |
| --- | --- | --- | --- | --- |
| 39 | HS-Oq-049 | 29.95718 | 47.94625 | Om Qaser |
| 40 | HS-Oq-050 | 29.95893 | 47.93938 | Om Qaser |
| 41 | HS-Oq-051 | 29.05912 | 47.46795 | Om Qaser |
| 42 | HS-Rq-053 | 30.05588 | 47.45823 | Al Ritqa |
| 43 | HS-Rq-054 | 30.05593 | 47.45647 | Al Ritqa |
| 44 | HS-Rq-055 | 30.05602 | 47.45415 | Al Ritqa |
| 45 | HS-Rq-056 | 30.05598 | 47.45373 | Al Ritqa |
| 46 | HS-Rq-057 | 30.05595 | 47.45235 | Al Ritqa |
| 47 | HS-Rq-058 | 30.05595 | 47.45055 | Al Ritqa |
| 48 | HS-Rq-059 | 30.05587 | 47.44775 | Al Ritqa |
| 49 | HS-Rq-060 | 30.05597 | 47.44555 | Al Ritqa |
| 50 | HS-Rq-061 | 30.05600 | 47.44323 | Al Ritqa |
| 51 | HS-Rq-062 | 30.05595 | 47.44038 | Al Ritqa |
| 52 | HS-Rq-063 | 30.05598 | 47.43920 | Al Ritqa |
| 53 | HS-Rq-064 | 30.05607 | 47.43845 | Al Ritqa |
| 54 | HS-Rq-065 | 30.05598 | 47.43750 | Al Ritqa |
| 55 | HS-Rq-066 | 30.05602 | 47.43587 | Al Ritqa |
| 56 | HS-Rq-067 | 30.05618 | 47.43378 | Al Ritqa |
| 57 | HS-Rq-068 | 30.05605 | 47.43163 | Al Ritqa |
| 58 | HS-Rq-069 | 30.05603 | 47.43012 | Al Ritqa |
| 59 | HS-Rq-071 | 30.05610 | 47.41717 | Al Ritqa |
| 60 | HS-Rq-073 | 30.06333 | 47.42717 | Al Ritqa |
| 61 | HS-Rq-074 | 30.08938 | 47.04482 | Al Ritqa |
| 62 | HS-Sh-082 | 29.10087 | 46.60533 | Al Shagaya |
| 63 | HS-Sh-083 | 29.12112 | 46.57105 | Al Shagaya |
| 64 | HS-Sh-084 | 29.12112 | 46.57105 | Al Shagaya |
| 65 | HS-Sh-085 | 29.12112 | 46.57105 | Al Shagaya |
| 66 | HS-Sh-087 | 29.12112 | 46.57105 | Al- Shagaya |
| 67 | HS-Sh-088 | 29.12112 | 46.57105 | Al Shagaya |
| 68 | HS-Sh-089 | 29.12112 | 46.57105 | Al Shagaya |
| 69 | HS-Sh-090 | 29.12112 | 46.57105 | Al Shagaya |
| 70 | HS-Sh-092 | 29.12112 | 46.57105 | Al Shagaya |
| 71 | HS-Sh-094 | 29.12112 | 46.57105 | Al Shagaya |
| 72 | HS-Sh-096 | 29.12112 | 46.57105 | Al Shagaya |
| 73 | HS-Sh-098 | 29.12112 | 46.57105 | Al Shagaya |
| 74 | HS-Sh-100 | 29.12112 | 46.57105 | Al Shagaya |

| 75 | HS-Sh-102 | 29.20330 | 46.66980 | Al Shagaya |
| --- | --- | --- | --- | --- |
| 76 | HS-Sh-103 | 29.20330 | 46.66980 | Al Shagaya |
| 77 | HS-Sh-104 | 29.20330 | 46.66980 | Al Shagaya |
| 78 | HS-Sh-106 | 29.20330 | 46.66980 | Al Shagaya |
| 79 | HS-Sh-107 | 29.20330 | 46.66980 | Al Shagaya |
| 80 | HS-Sh-109 | 29.20330 | 46.66980 | Al Shagaya |
| 81 | HS-Sh-111 | 29.20330 | 46.66980 | Al Shagaya |
| 82 | HS-Sh-112 | 29.28030 | 46.73088 | Al Shagaya |
| 83 | HS-Sh-113 | 29.28030 | 46.73088 | Al Shagaya |
| 84 | HS-Sh-115 | 29.28030 | 46.73088 | Al Shagaya |
| 85 | HS-Sh-118 | 29.28030 | 46.73088 | Al Shagaya |
| 86 | HS-Sh-120 | 29.28030 | 46.73088 | Al Shagaya |
| 87 | HS-Ab-121 | 29.35738 | 46.78903 | Al Abraq |
| 88 | HS-Ab-123 | 29.35738 | 46.78903 | Al Abraq |
| 89 | HS-Ab-125 | 29.35738 | 46.78903 | Al Abraq |
| 90 | HS-Ab-127 | 29.35738 | 46.78903 | Al Abraq |
| 91 | HS-Ab-129 | 29.35738 | 46.78903 | Al Abraq |
| 92 | HS-Ab-131 | 29.35738 | 46.78903 | Al Abraq |
| 93 | HS-Ab-133 | 29.50245 | 46.90935 | Al Abraq |
| 94 | HS-Ab-135 | 29.50245 | 46.90935 | Al Abraq |
| 95 | HS-Ab-136 | 29.50245 | 46.90935 | Al Abraq |
| 96 | HS-Ab-138 | 29.50245 | 46.90935 | Al Abraq |
| 97 | HS-Ay-139 | 29.55132 | 47.40373 | Al Abdally |
| 98 | HS-Ay-140 | 29.55416 | 47.40385 | Al Abdally |
| 99 | HS-Ay-141 | 29.56192 | 47.40166 | Al Abdally |
| 100 | HS-Ay-142 | 29.59441 | 47.39069 | Al Abdally |
| 101 | HS-Ay-143 | 30.00862 | 47.39075 | Al Abdally |
| 102 | HS-Ay-144 | 30.02928 | 47.40230 | Al Abdally |
| 103 | HS-Ay-145 | 30.04284 | 47.41495 | Al Abdally |
| 104 | HS-Ay-146 | 29.55915 | 47.41086 | Al Abdally |
| 105 | HS-Ay-147 | 29.56546 | 47.43758 | Al Abdally |
| 106 | HS-Ay-148 | 29.57111 | 47.48751 | Al Abdally |
| 107 | HS-Ay-149 | 29.58112 | 47.49986 | Al Abdally |
| 108 | HS-Ay-150 | 29.57471 | 47.49262 | Al Abdally |
| 109 | HS-Ay-151 | 29.56995 | 47.41862 | Al Abdally |
