# Supplementary material for: Genetic diversity and population structure of Haloxylon salicornicum moq. in Kuwait by ISSR markers
Source: PLoS One. 2018 Nov 21;13(11):e0207369. doi: 10.1371/journal.pone.0207369 (PMC6248962; doi:10.1371/journal.pone.0207369)
Supplement: S2 Table — (DOCX) [file pone.0207369.s004.docx]

| **S. No** | **Primer ID** | **Primer Sequence**  **(5ˈ-3ˈ)** | **Tm** |  | |
| --- | --- | --- | --- | --- | --- |
|  |  |  |  | **No. of Bands** | **Ann Temp. Temp. (**°C**)** |
| 1 | ISSR1 | (CA)_8_CG | 59.9 | 2 | 50 |
| 2 | ISSR2 | CA(GA)_7_GT | 57.6 | 11* | 55 |
| 3 | ISSR3 | (GA)_8_TC | 57.6 | 5* | 52 |
| 4 | ISSR4 | (CA)_8_TG | 57.6 | 3 | 45 |
| 5 | ISSR5 | (AAG)_6_GC | 56.3 | 5* | 52 |
| 6 | ISSR6 | (AAG)_6_TG | 54.2 | 0 | - |
| 7 | ISSR7 | (AGC)_6_GA | 66.6 | 7* | 50 |
| 8 | ISSR8 | (GGC)_6_TA | 76.8 | 5* | 60 |
| 9 | ISSR9 | GCG(ATT)_7_ | 49.2 | 2 | 45 |
| 10 | ISSR10 | CCCGGATCC(GA)_8_ | 69.5 | 8* | 65 |
| 11 | ISSR11 | CCCGGATCC(CT)_8_ | 69.5 | 5* | 65 |
| 12 | ISSR12 | CCCGGATCC(GT)_8_ | 69.5 | 7* | 60 |
| 13 | ISSR13 | CCCGGATCC(CA)_8_ | 69.5 | 6* | 60 |
| 14 | ISSR14 | GATC(CGG)_6_C | 83.5 | 1 | 72 |
| 15 | ISSR15 | GATCT(GGT)_6_GG | 71.1 | 4 | 60 |
| 16 | ISSR16 | GATCGAGGA(CGA)_5_C | 71.1 | 4 | 72 |
| 17 | ISSR17 | GATCCA(GCA)_6_C | 71.1 | 5* | 62 |
| 18 | ISSR18 | GAT(CAT)_8_C | 61.7 | 5* | 50 |
| 19 | ISSR19 | GATCT(TGT)_7_TG | 61.7 | 0 | - |
| 20 | ISSR20 | GA(TCT)_10_ | 63.4 | 2 | 60 |
| 21 | ISSR21 | GATCT(CTGT)_6_CTG | 69.8 | 1 | 60 |
| 22 | ISSR22 | GATCAGT(GAGT)_6_G | 69.8 | 0 | - |
| 23 | ISSR23 | (GA)_11_A | 62.8 | 5* | 50 |
| 24 | ISSR809 | (AG)_10_G | 62.6 | 7* | 52 |
| 25 | ISSR810 | (GA)_8_T | 54.8 | 6* | 50 |
| 26 | ISSR811 | (GA)_8_C | 57.2 | 3 | 57 |

| **S. No** | **Primer ID** | **Primer Sequence**  **(5ˈ-3ˈ)** | **Tm** |  | | | |  |
| --- | --- | --- | --- | --- | --- | --- | --- | --- |
|  |  |  |  | **No. of Bands** | | **Ann Temp. Temp. (**°C**)** |  |  |
| 27 | ISSR815 | (ACG)_8_AC | 72.5 | 4 | | 62 |  |  |
| 28 | ISSR820 | (AC)_9_C | 60.2 | 6* | | 60 |  |  |
| 29 | ISSR826 | (AC)_8_AG | 57.6 | 5* | | 45 |  |  |
| 30 | ISSR827 | (AC)_9_CG | 62.4 | 2 | | 57 |  |  |
| 31 | ISSR828 | (TG)_8_GA | 54.8 | 0 | | - |  |  |
| **Total primers screened** | | | | | **16** | | | |

**(*** Primers were used further for ISSR analysis)
